# Cell cycle arrest is not senescence

**DOI:** 10.18632/aging.100281

**Published:** 2011-02-06

**Authors:** Mikhail V. Blagosklonny

**Affiliations:** Department of Cell Stress Biology, Roswell Park Cancer Institute, Buffalo, NY, 14263, USA

**Keywords:** Cellular senescence, locked quiescence, growth stimulation, mTOR, rapamycin, gerossuppressants

## Abstract

DNA damaging agents and radiation, cytotoxins and anti-cancer drugs, telomere erosion and cytokines, culture shock and mitogenic stimuli, oncogenes and tumor suppressors can induce both cell cycle arrest and cellular senescence. Due to this semi-coincidence, senescence is confused with cell cycle arrest, or even more misleadingly, with growth inhibition. With such misconceptions, cellular senescence cannot be linked to organismal aging. Also, the relation between cancer and senescence is distorted. Here I discuss why the link between arrest and senescence is semi-coincidental and how senescence is related to aging and cancer.

## Quiescence versus senescence

**Figure 1.**
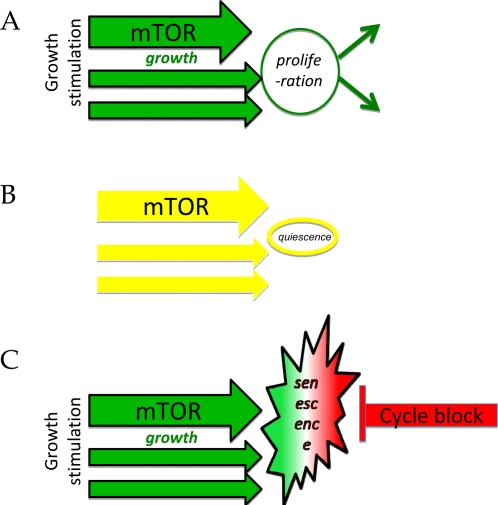
Two types of cell cycle arrest. (**A**) Proliferating cells. Growth stimulation leads to mass growth, which is balanced by cell division. (**B**) Quiescence. Withdrawal of growth factors deactivates both growth-promoting pathways and the cell cycle. (**C**) Senescence. The block of the cell cycle, in the face of growth-stimulation, causes condition known as cellular senescence.

In the adult organism, most cells are arrested but they are not senescent. So cell cycle arrest is not a synonym of senescence. Non-senescent arrest can be caused by withdrawal of serum growth factors and nutrients (Figure 1A versus B). Without growth factors, cells become quiescent: low metabolism, protein synthesis and cellular functions, no cellular size growth. Consider an analogy. You are driving a car, pushing the gas pedal (analogous to growth stimulation). Then you release the gas pedal (an equivalent to serum withdrawal), the car decelerates and stops. This is quiescence, a reversible arrest.

But withdrawal of growth factors is not the only way to arrest cell cycle. Induction of CDK inhibitors such as p21, p16, p57 can cause cell cycle arrest in the presence of serum (Figure 1C). Serum growth factors, hormones, high levels of nutrients and oxygen stimulate growth-promoting pathways such as MAPK (mitogen-activated protein kinase) and mTOR (Target of Rapamycin) pathways [1,2]. (Furthermore, cancer cells have constitutively over-activated by mutations mTOR and MAPK pathways). While blocking the cell cycle, CDK inhibitors do not deactivate growth-promoting pathways such as mTOR and MAPK. In other words, while growth is stimulated, cell cycle is blocked (Figure 1C). By analogy, this is like pushing the gas and hitting the brakes simultaneously, with an increasing force. This is destructive.

In theory [3,4], over-activated growth-promoting pathway, when the cell cycle is blocked downstream, must lead to cellular hypertrophy (a large cell morphology), pro-inflammatory and hyper-secretory phenotypes, cellular overactivation with a feedback signal-resistance and a compensatory deactivation of some signaling pathways. Cellular hypertrophy will cause compensatory activation of lysosomes, autophagy (despite active mTOR) and beta-Gal-positivity. This theoretical condition strikingly resembles senescence caused by DNA damaging agents and radiation, mitogenic stimuli, oncogenes and tumor suppressors [5-9], which all induce CDK inhibitors, thus blocking the cell cycle despite continuous growth stimulation (Figure 1C). Pushed by growth-stimuli, senescent cells simultaneously have high levels of CDK inhibitors and cyclins D and E [10-13]. Erroneously, it is commonly repeated that senescence is an “exit from the cell cycle”. In reality, it is an active arrest in very advanced points of G1, G1/S and even G2. The senescent cell is driven to cycle by the stuck accelerator pedal but is blocked by the powerful brakes. The tension is manifested as pseudo-DNA-damage response, an atypical response without detectable DNA damage [14], perhaps similar to a chronic atypical response, described as DNA-SCARS [15]. Senescent cells secrete both mitogenic and anti-mitogenic factors [16-27].

The conflict between ‘acceleration and braking’ leads to inappropriate S-phase entry and, on the other hand, to the loss of proliferative potential (PP). PP is not proliferation, PP is a potential, a hidden quality of arrested cells. The only way to measure PP is to remove the brakes. For example, ectopic expression of p21 causes arrest, which becomes irreversible after 3-4 days, meaning that cells cannot proliferate even after removal of p21 [28,29]. Loss of PP defines cellular senescence in cell culture, distinguishing it from reversible quiescence. Still this does not imply that loss of PP is a clinically relevant marker.

## Cellular senescence in vitro and in the organism

*In vitro*, cellular senescence is defined by the loss of proliferative potential (PP). Loss of PP seems to be one of consequences of cellular overactivation and correlates with cellular hypertrophy [29]. This marker is universal: all senescent cells - fibroblasts and epithelial cells, either normal or malignant - share this marker. This is convenient. However, this marker is not the most important for organismal aging [30]. From the medical perspective, a single most important marker of cellular senescence is increased cellular functions (hyper-functions). At first, this statement may seem startling, because hyper- functions were not considered as markers of senescence. Or were they? Most studies of senescence were performed in fibroblasts and tumor cells of fibroblast origin. The classic function of such cells is secretion. And hyper-secretory phenotype is a well-known marker of senescence; a marker that, by the way, links cellular senescence to organismal aging and cancer [17-22,31]. Cellular functions are tissue-specific: contraction for smooth muscle cells, secretion of lipoproteins for hepatocytes, aggregation for platelets, oxidative burst for neutrophils, bone resorption for osteoclasts and so on. These hyper-functions lead to age-related diseases, such as atherosclerosis, hyper-tension, macular degeneration, increasing the probability of organismal death [32,33].

**Figure 2.**
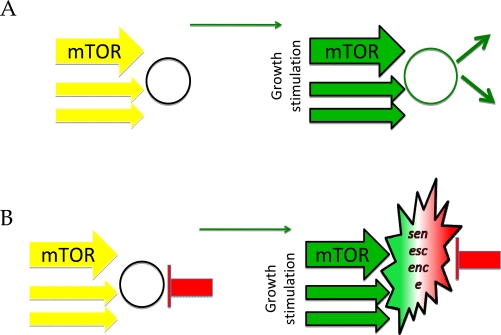
Two types of quiescence. (**A**) Simple quiescence. Cells are arrested due to lack of growth stimulation (left panel). Addition of growth factors causes proliferation (right panel). (**B**) Locked quiescence. Differentiated cells are put on the brakes, to avoid undesired proliferation. Mild stimulation of such cells causes functional responses. Excessive stimulation causes physiological senescence.

In cell culture, quiescence could be imitated by serum withdrawal. (Figure 2A) Then re-stimulation leads to proliferation (Figure 2A, right panel). In the organism, most of the cells are arrested but not senescent. Stimulation can cause their proliferation. Examples may include some (but not all) fibroblasts, lymphocytes, stem and satellite cells. In quiescent stem cells, over-activation of the mTOR pathway causes stem cell proliferation and exhaustion [34-38].

In the organism, “for safety”, quiescent cells could be put on a permanent “parking brake”: an arrest locked by CDK inhibitors. Perhaps, adipocytes, neurons, cardiomyocytes can serve as examples. In locked cells, stimulation increases cell functions, instead of proliferation. For example, adipocytes will accumulate fat, whereas cardiomyocytes will enlarge and endocrine cells will secrete. Over-stimulation can cause cellular hyper-functions, secondary hormone/stimuli resistance and even cell loss. This chronic over-stimulation of initially quiescent cells could be called physiological senescence. In the organism, differentiated post-mitotic cells undergo physiological senescence due to chronic over-activation.

Physiological senescence can be modeled in cell culture. Serum withdrawal arrests normal cells. Then these quiescent cells can be additionally put on brakes: a condition we named locked quiescence [39]. Then re-addition of serum stimulates growth in size (hypertrophy), senescent morphology and permanent loss of PP. It was shown that differentiated cells, especially in the organism, are indeed locked by CDK inhibitors [40,41]. In theory, such cells could still be quiescent or senescent. Over-stimulation of growth-promoting pathways (such as mTOR) converts ‘locked’ quiescence into senescence [39], a process that models physiological senescence. From cell culture models to the organism, it is stimulation of growth-promoting pathways rather than cell cycle arrest *per se* that determines senescence.

**Figure 3.**
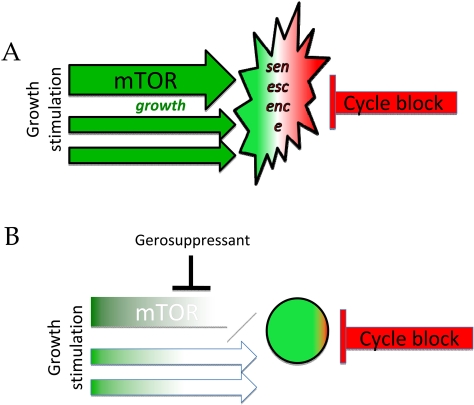
Gerosuppressants favor quiescence over senescence by inhibiting growth-promoting pathways. (**A**) Senescent cell. (**B**) Gerossuppressants do not abrogate arrest but suppress the senescent phenotype converting senescence in locked quiescence.

## Cell cycle arrest and cancer

The most common introductory statement about senescence is that it is a barrier to cancer. However, it is cell cycle arrest that is a barrier to cancer. In fact, avoidance of arrest is a common alteration in cancer. And an even more common alteration is the activation of growth-promoting pathways such as MAPK and mTOR, which are involved in the senescent phenotype. Activation of MAPK and mTOR makes cancer cells pro-senescent: it is sufficient to impose cycle arrest in order to reveal the senescent phenotype. The pro-senescent phenotype due to overactivation of MAPK and PI3K/mTOR can be linked to hallmarks of cancer such as angiogenesis, apoptosis-avoidance, Warburg effect, invasion and metastasis (I will discuss this in forthcoming reviews). If so, then the pro-senescent phenotype determines 4 out of 6 hallmarks of cancer (see 6 hallmarks of cancer by Hanahan and Weinberg [42]. Therefore, cancer depends on both the pro-senescent phenotype and the disabled cell cycle control. I suggest that cell cycle arrest typically leads to senescence in cancer because cancer is a pro-senescent state (over-activation of mTOR-centric network) and cell cycle arrest simply allows its manifestation.

## Tumor suppressors, gerosuppressors and gerosuppressants

Some tumor-suppressors (TS) such as Rb and p16 cause cell cycle arrest. Other TS such as PTEN and TSC1/2 inhibit the growth-promoting mTOR pathway, which is involved in the pro-senescent phenotype. An ultimate tumor suppressor would have both activities: (a) cause arrest (which is a barrier in cancer) and (b) suppress the pro-senescent phenotype. In fact, such a tumor-suppressor is p53 [43-50]. Suppression of the senescent phenotype by p53 may be in part explained by the inhibition of mTOR and hyper-metabolism by p53 [51-59]. The notion that p53 suppresses senescence may also explain life extension by p53 [60]. (Note: Deletion of senescence-suppressing TS such as PTEN, TSC1/2 and VHL can lead to premature senescence. In comparison, deletion of p53 bypass the senescence, because loss of p53 simultaneously abrogates cell cycle arrest. This leads to cancer: proliferating pro-senescent cells. I will address this topic in detail in my future reviews).

Similarly, rapamycin suppresses the senescent phenotype. In cells arrested by p21, rapamycin decelerates the conversion from locked quiescence to senescence. Thus, rapamycin and other inhibitors of mTOR can preserve PP in p21-arrested cells [13,29,61-63]. Please do not misunderstand this as the abrogation of cycle arrest and cancer-promotion. The terms proliferation and proliferative *potential*(PP) should not be confused. Rapamycin does not decrease p21, does not prevent cell cycle arrest caused by p21, does not ‘unlock’ cells, does not force cells to proliferate, of course. In contrast, it can inhibit proliferation on its own. But in p21-arrested cells, rapamycin can preserve the potential to proliferate (PP). Only when p21 and rapamycin are removed, the potential can be determined. Rapamycin does not “suppress” cell cycle arrest. Rapamycin delays the conversion of arrest into senescence. In some cell types, rapamycin can cause cell cycle arrest. But while inhibiting proliferation, rapamycin preserves PP.

I put emphasis on the preservation of PP by rapamycin (rather than, for example, on the suppression of the hyper-secretory phenotype, which rapamycin also inhibits), simply because PP is viewed as a definitive marker of senescence. Therefore, rapamycin is a gerosuppressant by the current definition of cellular senescence [64]. However, it is suppression of other markers of senescent phenotype such as hyper-secretion and other hyper-functions that are most clinically relevant.

By simultaneously suppressing the senescent phenotype and causing arrest, rapamycin can be viewed as an ultimate tumor-suppressant. In fact, the hyper-secretory, pro-inflammatory, pro-angiogenic phenotype are markers of both senescence and cancer. I suggest that the cancer-preventive effect of rapamycin [65] is not because (or not only because) of cell cycle arrest but because of suppression of the senescent phenotype, especially in normal cells.

## CONCLUSIONS

**Figure 4.**
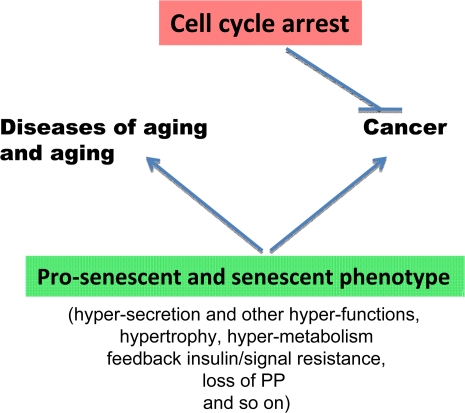
The opposite roles of senescence and cell cycle arrest. Cell cycle arrest is a barrier to cancer. In contrast, cellular senescence promotes cancer and age-related diseases.

Cell cycle arrest (the good half) is only a part of the equation of senescence. The second part is growth stimulation, which actually causes the senescent phenotype (the bad half). While cell cycle arrest is a barrier to cancer, senescence (in both cancer and normal cells) is a prerequisite for cancer (Figure 4). This extends the notion that the secretory phenotype contributes to cancer and that cancer and aging have a lot in common [22,66-68]. Furthermore, I suggest that all hallmarks of senescence together, especially an increase in tissue-specific cellular functions, caused by cellular over-stimulation lead to all age-related diseases (organismal aging) (Figure 4).

So cell cycle arrest is not senescence. In cell culture, cell cycle arrest typically leads to senescence, because the cell is over-stimulated by serum, nutrients, oncogenes and so on. Therefore, cell cycle arrest is sufficient to cause senescence, especially in cancer cells. This is why arrest of cell cycle is semi-coincidentally confused with senescence. Senescent phenotype can be dissociated from cycle arrest. And gerosuppressants can suppress the senescent phenotype (including loss of PP) without abrogating (and even increasing) arrest.
